# Overexpression of hindsight in sensory organ precursors is associated with a transformation of campaniform sensilla to microchaetae in the Drosophila wing

**DOI:** 10.17912/micropub.biology.000103

**Published:** 2019-05-20

**Authors:** Krzysztof Szablewski, Bruce H Reed

**Affiliations:** 1 Department of Biology, University of Waterloo, Waterloo, ON, Canada N2L 3G1

**Figure 1.**
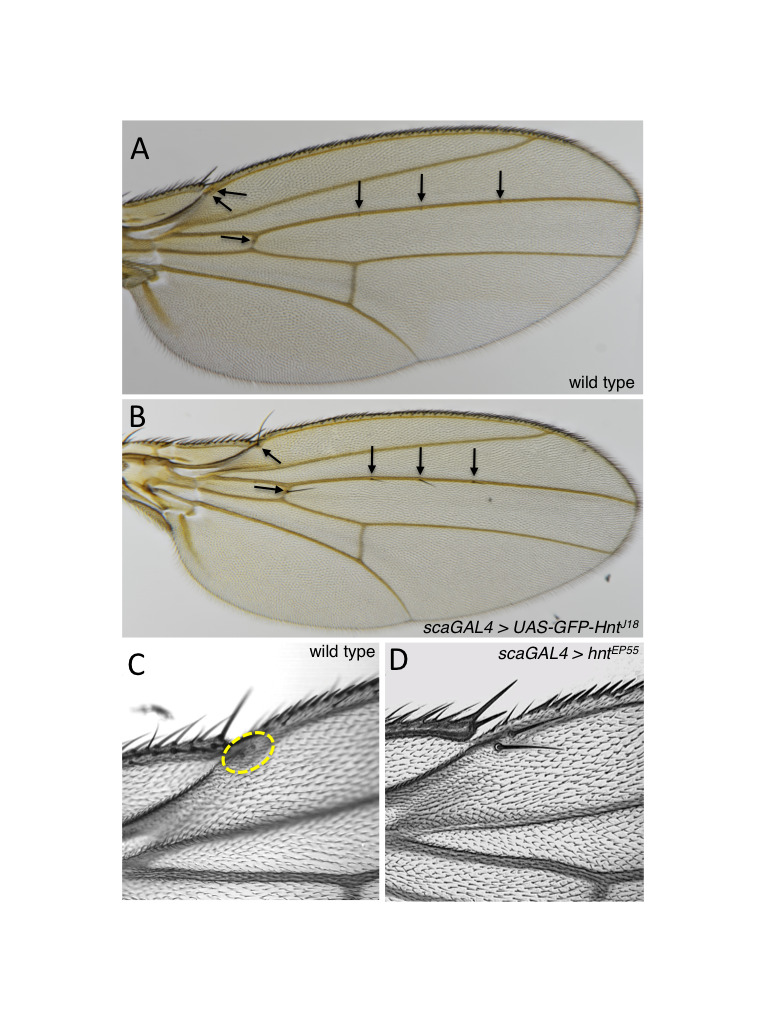
Overexpression of *hnt* results in a transformation of campaniform sensilla to microchaetae. (A) Wild type wing showing stereotypical positions of campaniform sensilla on the dorsal surface of the wing blade (arrows). (B) Wing of *scaGAL4* > *UAS-GFP-hnt[J18]* showing a transformation of five campaniform sensilla to microchaetae (arrows). (C) Wild type wing showing twin campaniform sensilla located on the first longitudinal vein (dashed yellow circle). (D) Wing of *scaGAL4* > *peb[EP55]* showing microchaetae located in the normal positions of the twin campaniform sensilla.

## Description

The adult Drosophila wing blade contains sense organs known as campaniform sensilla. These cellular structures sense pressure or strain in the wing during flight and provide neural feedback required for coordinated wing movements (Bartussek and Lehmann, 2016). The dorsal surface of the wing blade includes three campaniform sensilla along wing vein L3, two on the first longitudinal vein near the tip of the costa, and a single sensillum on the anterior cross vein. Other campaniform sensilla are found at the base of the wing and on the ventral wing surface (Huang et al., 1991). In experiments utilizing GAL4/UAS inducible expression of the Zinc finger transcription factor *hindsight* (*hnt*), also known as *pebbled* (*peb*) (Yip et al., 1997), we found that campaniform sensilla are frequently transformed to mechanosenory external sense organs, known as microchaetae. The GAL4 driver used was *scaGAL4*, a reporter driving expression of GAL4 in the pattern of the proneural gene *scabrous* (*sca*), which is expressed in sensory organ precursor cells as is *hnt* (Buffin and Gho, 2010). We found campaniform to microchaetae transformation occurred in the context of *scaGAL4* driving expression of a particular transgene insertion, *UAS-GFP-hnt[J18]*. In order to rule out the possibility that this transformation was somehow specific to *UAS-GFP-hnt[J18]*, we also tested *scaGAL4* > *peb[EP55]* and found the same transformation. The penetrance of this phenotype was not complete, as not all campaniform sensilla were transformed to microchaetae in all individuals. Approximately half of progeny carrying *scaGAL4* and either *peb[EP55]* or *UAS-GFP-hnt[J18]* display at least one transformation. To the best of our knowledge, there are two instances of this particular phenotype reported in the literature. The first is a specific allelic combination of loss-of-function mutants of the gene *absent, small, or homeotic discs 2* (*ash2[7]/ash2[18]*) (Adamson and Shearn, 1996). The second involves the expression throughout the wing imaginal disc of a human SMAD tumour allele (*UAS-SMAD4[100T]*) (Takaesu et al., 2005). The former instance involving *ash2* loss-of-function mutants is of particular interest because *ash2* is implicated in the negative regulation of EGFR/Ras/MAPK signalling, through the negative regulation of *rhomboid* (Angulo et al., 2004), which is required for the production of active EGFR ligand (Wasserman et al., 2000). Thus, overexpression of *hnt* is consistent with overactivation of EGFR/Ras/MAPK signalling in the context of campaniform sensilla SOPs. Interestingly, *hnt* is the Drosophila homologue of human *Ras Responsive Element Binding protein-1* (*RREB-1*) (Melani et al., 2008; Ming et al., 2013).

## Reagents

The *scaGAL4* (y w[*]; P{w[+mW.hs]=GawB}sca[109-68]/CyO) and *peb[EP55]* (*w[1118] P{w[+mC]=EP}peb[EP55]*) lines are available from the Bloomington Drosophila Stock Center (Bloomington stocks 6479 and 5358, respectively). The *UAS-GFP-hnt[J18]* line was recovered as follows: the original *UAS-GFP-hnt* insertion (Ming et al., 2013), which maps to the second chromosome, was mobilized by crossing to a *Delta2-3 P* element transposase stock (*y w; ry[506] Sb P{ry[+t7.2]=Delta2-3}99B/TM6*). F1 progeny were subsequently crossed to *y w[1118]*. Single F2 males lacking the *Sb* *Delta2-3* chromosome and whose eye color differed substantially from that of the original *UAS-GFP-hnt* line, were isolated and propagated as stable stocks. Insertions mapping to the second or third chromosome were maintained as balanced or homozygous stocks and tested for *GFP-hnt* expression by crossing to the eye specific *GMR-GAL4* driver (*P{w[+mC]=GAL4-ninaE.GMR}12,* Bloomington stock 1104). While the original *UAS-GFP-hnt* line was pupal lethal with *GMR-GAL4*, *UAS-GFP-hnt[J18]* produced viable progeny with a rough eye phenotype.

Wings were removed from anesthetized flies and placed in a drop of xylene on a microscope slide. Most xylene was then removed by touching the corner of a folded piece of tissue paper to the drop of xylene, leaving the wings in a thin film of xylene. A drop of Permount mountant (Fisher Scientific) was placed over the wings and a coverslip was placed over the wings in the mountant. Color wing micrographs were taken using a Nikon SMZ25 stereomicroscope equipped with Nikon Digital Sight Ri2 16.25MP color camera and processed using extended-depth-of-focus feature of Nikon NIS-Elements Arv4.50 software. The black and white images were obtained using a Nikon Eclipse 90i fitted with a Nikon D-eclipse C1 scan head using Nikon EZ-C1 software and brightfield detection. Brightfield Z stacks were edited and assembled using ImageJ (Schneider et al., 2012) and an extended-depth-of-focus plugin.
